# The effects of coagulation factors and their inhibitors on proliferation and migration in colorectal cancer

**DOI:** 10.1002/cam4.6332

**Published:** 2023-07-17

**Authors:** Peter Adam Rees, John Castle, Hamish William Clouston, Rebecca Lamb, Urvashi Singh, Sarah Elizabeth Duff, Cliona Clare Kirwan

**Affiliations:** ^1^ Division of Cancer Sciences, School of Medical Sciences, Faculty of Biology, Medicine and Health University of Manchester Manchester UK; ^2^ Manchester University NHS Foundation Trust Manchester UK; ^3^ The Christie NHS Foundation Trust Manchester UK; ^4^ Department of Life Sciences Manchester Metropolitan University Manchester UK

**Keywords:** coagulation factors, colorectal cancer, migration, proliferation

## Abstract

**Background/Aim:**

Clotting factors promote cancer development. We investigated if coagulation proteins promote proliferation and migration in colorectal cancer (CRC) cell lines and whether their direct inhibitors can attenuate these effects.

**Materials and Methods:**

DLD‐1 and SW620 cells were treated with tissue factor (0, 50, 100 and 500 pg/mL ± 10 μg/mL 10H10 [anti‐tissue factor antibody]), thrombin (0.0, 0.1, 1.0 and 10.0 U/mL ± 0.5 μM dabigatran [thrombin inhibitor]) and Factor Xa, FXa (0.0, 0.1, 1.0 and 10.0 U/mL ± 100 ng/mL rivaroxaban [FXa inhibitor]) and their effects on proliferation and migration were quantified using the PrestoBlue® and transwell migration assays, respectively.

**Results:**

Thrombin increased proliferation from 48 h treatment compared to its control (48 h 6.57 ± 1.36 u vs. 2.42 ± 0.13 u, *p* = 0.001, 72 h 9.50 ± 1.54 u vs. 4.50 ± 0.47 u, *p* = 0.004 and 96 h 10.77 ± 1.72 u vs. 5.57 ± 0.25 u, *p* = 0.008). This increase in proliferation was attenuated by dabigatran at 72 h (2.23 ± 0.16 u vs. 3.26 ± 0.43 u, *p* = 0.04).

Tissue factor (0 pg/mL 20.7 ± 1.6 cells/view vs. 50 pg/mL 32.4 ± 1.9 cells/view, *p* = 0.0002), FXa (0.0 U/mL 8.9 ± 1.1 cells/view vs. 10.0 U/mL 17.7 ± 1.7 cells/view, *p* < 0.0001) and thrombin (0.0 U/mL 8.9 ± 1.3 cells/view vs. 10.0 U/mL 20.2 ± 2.0 cells/view, *p* < 0.0001) all increased migration compared to their controls. However, their direct inhibitors did not attenuate these increases.

**Conclusion:**

Thrombin, FXa and TF all increase migration in CRC in vitro. Thrombin induced increase in proliferation is abrogated by dabigatran. Dabigatran may have potential as an anti‐cancer therapy in CRC.

## Graphical abstract

We investigated if coagulation proteins promote proliferation and migration in colorectal cancer (CRC) cell lines, and whether their direct inhibitors can attenuate these effects. Thrombin, factor Xa and tissue factor all increase migration in CRC in vitro. A thrombin‐induced increase in proliferation is abrogated by dabigatran.

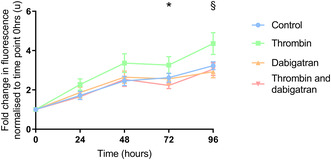

## 1 INTRODUCTION

Malignancy is associated with a hypercoagulable state, resulting in the clinical manifestation of venous thromboembolism (VTE). The risk of symptomatic VTE is increased sevenfold in patients with cancer.^1^, ^2^ Colorectal cancer (CRC) is the second most common cause of cancer death in the United Kingdom, with about 16,800 deaths each year.^3^ Approximately 5% of CRC patients will develop a VTE.^4^ The risk is greatest in more advanced cancers and those undergoing thrombogenic therapies, including surgery and chemotherapy.^4^

VTE results from the pathological activation of the coagulation system. Tissue factor (TF), the initiator of the coagulation system, sets in motion a series of cellular‐based reactions involving the key downstream effectors, factor Xa (FXa) and thrombin. This ultimately leads to the production of a fibrin clot.^5^ The direct oral anticoagulants dabigatran and rivaroxaban, which inhibit thrombin and FXa, respectively, are in routine clinical use in VTE prophylaxis.

The symbiotic relationship between CRC and the extrinsic clotting pathway extends beyond an increased risk of VTE. Tissue factor (TF), the initiator of the extrinsic clotting pathway has increased expression in CRC^6^, ^7^, ^8^ and its expression correlates with change in *KRAS* and *TP53* mutational status in CRC cell lines.^9^ TF and thrombin promotion of cancer processes could be achieved by binding to and activating specific G‐protein‐coupled receptors, that is, protease‐activated receptors (PAR)‐1 and ‐2.^10^ In in vitro studies, the over‐expression of TF and PAR‐2 in a CRC cell line led to increased proliferation and motility,^11^ possibly via PKCα and ERK1/2 signalling pathways.^12^, ^13^ The pro‐proliferative effects of epithelial TF expression in epithelial CRC cell lines has been demonstrated in multiple cell lines.^14^ PAR‐1 activation by thrombin may have a pro‐migratory effect in cancer by affecting the expression of the αvβ5 integrin^15^; however, this is not consistent in the literature.^16^

Despite the previous focus on *epithelial* expression of extrinsic clotting factors, key coagulation factors, including TF, have been shown to be upregulated in the tumour microenvironment (TME) in breast cancer.^17^ Furthermore, PAR‐1 and PAR‐2 are also expressed by cancer‐associated fibroblasts (CAFs).^18^ It is possible that *exogenous* extrinsic clotting factors from the TME may influence tumour biology.

In a recent study by Graf et al., rivaroxaban promoted anti‐cancer immunity in colorectal cancer murine models.^19^ Indeed, the use of anticoagulants to improve outcomes in cancer is not a new concept. As early as the 1980s improved survival was described in patients with small‐cell lung cancer who were treated with warfarin.^20^ Conflicting effects of low molecular weight heparin (LMWH) on survival in cancer have been observed.^21^, ^22^, ^23^ However, neither warfarin nor LMWH exert their effects via the TF/FVIIa/PAR‐2 axis which appears to be fundamental to cancer progression. Pharmacologic inhibition of hepatic prothrombin production with a non‐clinically approved agent impedes murine and human colon cancer growth in vivo.^24^ There is also new therapeutic interest in targeting colorectal cancer TF expression, including with the antibody‐drug conjugate tisotumab vedotin that is currently undergoing Phase 2 trials for efficacy and safety.^25^ A monoclonal antibody to TF, 10H10 (Clone: TF9‐10H10), is commercially available although is not approved for clinical use.

The aim of this study was to investigate the effects of exogenous extrinsic clotting pathway factors, that is, thrombin, FXa and TF, on the key cancer processes of proliferation and migration in CRC cells in vitro and determine if these effects can be mitigated by the addition of their direct inhibitors.

## 2 MATERIALS AND METHODS

### 2.1 Cell lines and culture

Human colorectal cancer cell lines DLD‐1 and SW620 were used for all assays (American Type Culture Collection [ATCC]). DLD‐1 cells were maintained in McCoy's 5A modified medium (ThermoFisher Scientific) supplemented with 10% foetal bovine serum (FBS, Gibco, ThermoFisher Scientific), 100 U/mL penicillin G and 100 μg/mL streptomycin sulphate (ThermoFisher Scientific). SW620 cells were maintained in Dulbecco's modified Eagle medium (DMEM, Sigma‐Aldrich) supplemented with 10% FBS (Gibco, ThermoFisher Scientific), 100 U/mL penicillin G and 100 μg/mL streptomycin sulphate (ThermoFisher Scientific) and 2 mM GlutaMAX (ThermoFisher Scientific). Cells were cultured at 37°C and 5% CO_2_/air.

### 2.2 Cell line authentication

Cell lines were authenticated by STR (short tandem repeat) analysis using the Promega® Powerplex 21 System in the Molecular Biology Core Facility at the Cancer Research UK Manchester Institute.

### 2.3 Reagents

The human coagulation factors thrombin (Sigma‐Aldrich), factor Xa (Enzyme Research Laboratories) and recombinant coagulation factor III/tissue factor (R&D Systems) were purchased from the manufacturers.

Their respective inhibitors dabigatran (Boehringer Ingelheim), rivaroxaban (Bayer) and 10H10, a monoclonal antibody to TF, (Clone TF9‐10H10, Abcam) were similarly obtained.

The cell viability reagent PrestoBlue® was obtained from ThermoFisher Scientific.

### 2.4 Proliferation assay

Proliferation was quantified using the PrestoBlue® proliferation assay. Cells were seeded at a density of 2.5 × 10^3^ (DLD‐1) or 5.0 × 10^3^ (SW620) cells per well in 180 μL of complete cell line‐specific media in a 96‐well plate (Black with clear flat bottom). Three wells were plated per experimental condition. Wells containing cell line‐specific media only were used to correct for background fluorescence. After 24 h the media was replaced with complete, cell line‐specific media supplemented with exogenous coagulation factors ± direct inhibitors/monoclonal antibodies at physiological relevant doses (tissue factor 0, 50, 100 and 500 pg/mL, thrombin 0.0, 0.1, 1.0 and 10.0 U/mL ± 0.5 μM dabigatran and FXa 0.0, 0.1, 1.0 and 10.0 U/mL ± 100 ng/mL rivaroxaban). PrestoBlue® (20 μL) was added at time points 0, 24, 48, 72 and 96 h and, following incubation at 37°C for 1 h, fluorescence was measured using a FLUOstar Omega microplate reader (BMG Labtech). Appropriate vehicle controls were used throughout.

### 2.5 Migration assay

Migration was quantified using the transwell migration assay. Cell culture inserts (8.0 μm pores) were placed in empty 24 well plates and the upper chamber was primed with 200 μL of serum‐free media at 37°C. After 30 min this was replaced with 5.0 × 10^4^ DLD‐1 cells suspended in 200 μL serum‐free, cell line‐specific media containing exogenous coagulation factors ± direct inhibitors (tissue factor 0, 50, 100 and 500 pg/mL ± 10 μg/mL 10H10, thrombin 0.0, 0.1, 1.0 and 10.0 U/mL ± 0.5 μM dabigatran and FXa 0.0, 0.1, 1.0 and 10.0 U/mL ± 100 ng/mL rivaroxaban). About 600 μL of complete media supplemented with 10% FBS was added to the lower chamber as a chemoattractant. Following overnight incubation at 37°C, non‐migratory cells were removed with a cotton bud and washed with phosphate‐buffered saline. Migrated cells on the underside of the membrane were stained with crystal violet (Sigma‐Aldrich). After 20 min, excess stain was removed and the inserts were allowed to dry overnight. The number of migrated cells were manually counted over nine random fields per well at 200× magnification using light microscopy by a single scorer. Appropriate vehicle controls were used throughout.

### 2.6 Statistical analysis

All experiments were performed in triplicate across three independent experiments. Statistical differences between continuous variables were determined using the independent Student's *t*‐test or analysis of variance (ANOVA). *p* values <0.05 were considered significant. Statistical analysis was performed using GraphPad Prism version 7.0 (GraphPad Software).

## 3 RESULTS

In this study the exogenous coagulation factors thrombin, FXa and TF were added to the colorectal cancer cell lines SW620 and DLD‐1 to assess their effects on proliferation and migration in vitro. The datasets generated and analysed in this research article are available in the Figshare repository.^26^

### 3.1 Proliferation

**FIGURE 1:**
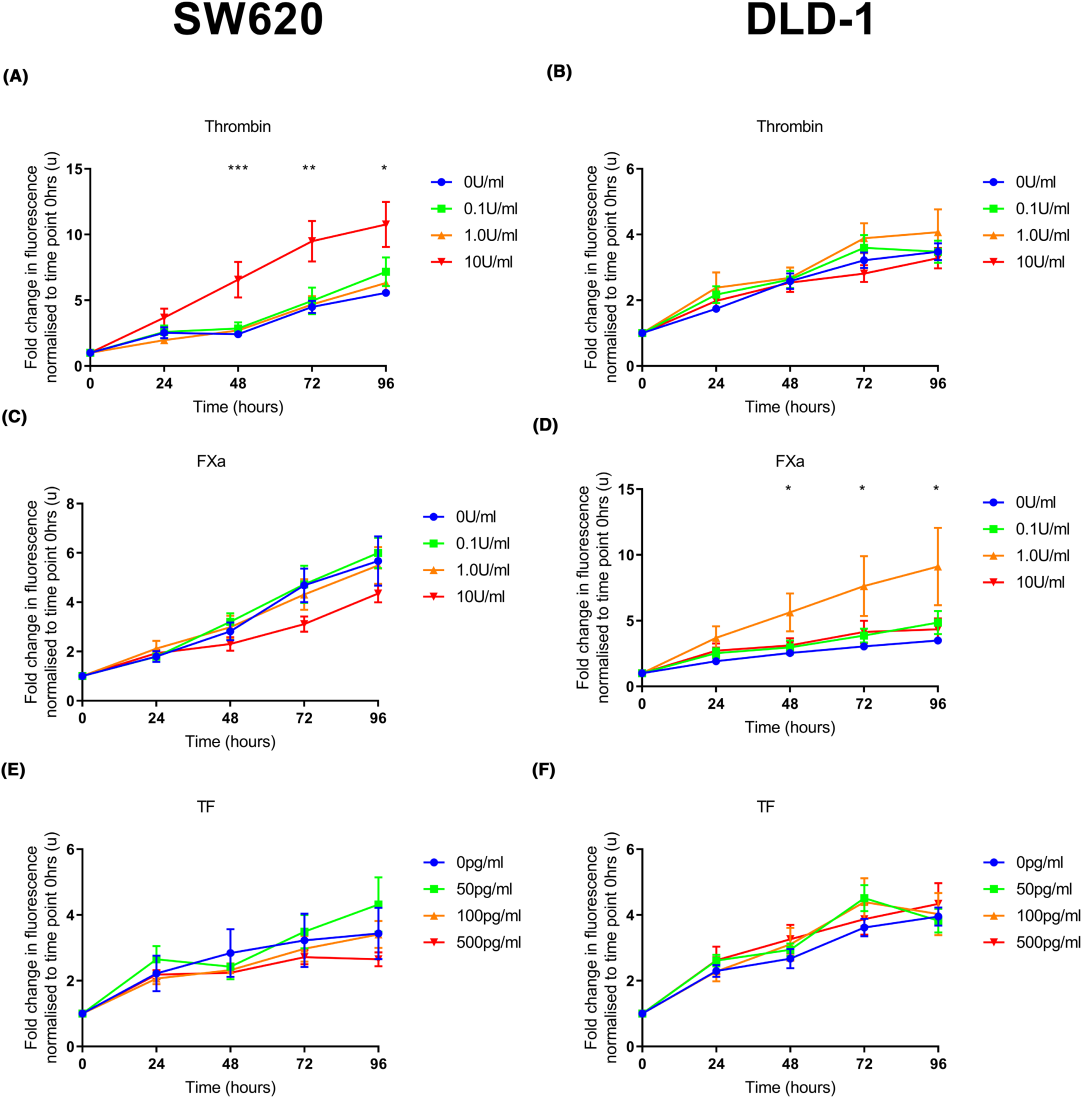
The effect of exogenous coagulation factors on proliferation in colorectal cancer cell lines. SW620 or DLD‐1 cells were seeded (5.0 × 10^3^ and 2.5 × 10^3^ cells per well, respectively). Exogenous clotting factors were added at 24 h (thrombin 0.0–10 U/mL, FXa 0.0–10 U/mL or TF 0–500 pg/mL). PrestoBlue® was added at intervals and fluorescence was measured. (A) Thrombin (10 U/mL) increased proliferation from 48 h onwards compared to the vehicle control in the SW620 cell line. There was no difference in proliferation at lower concentrations of thrombin. (C, E) There was no difference in proliferation in the SW620 cell line following the addition of FXa or TF at any concentration compared to the vehicle control. (B, F) There was no difference in proliferation in the DLD‐1 cell line following the addition of thrombin or TF at any concentration compared to the vehicle control. (D) FXa (1.0 U/mL) increased proliferation from 48 h onwards compared to the vehicle control in the DLD‐1 cell line. There was no difference at other concentrations of FXa. Data presented as mean fold change in fluorescence normalised to time point 0 h ±SEM. Data from at least three independent experiments. Statistical differences were determined using analysis of variance (ANOVA). *p < 0.05, **p < 0.01, ***p < 0.001. TF, tissue factor.

Thrombin at 10 U/mL increased proliferation of SW620 cells from 48 h onwards compared to vehicle control (Figure 1A). Thrombin at lower concentrations did not affect proliferation and did not affect proliferation in the DLD‐1 cell line (Figure 1B).

FXa had no effect on proliferation in the SW620 cell line (Figure 1C), but increased proliferation of DLD‐1 cells at a concentration of 1.0 U/mL from 48 h onwards (Figure 1D).

Exogenous TF had no effect on proliferation at the 50–500 pg/mL concentration range, which was chosen to reflect reported colorectal cancer patient plasma levels.^27^ We did not increase the concentration of TF administered beyond this (Figure 1E,F).

**FIGURE 2:**
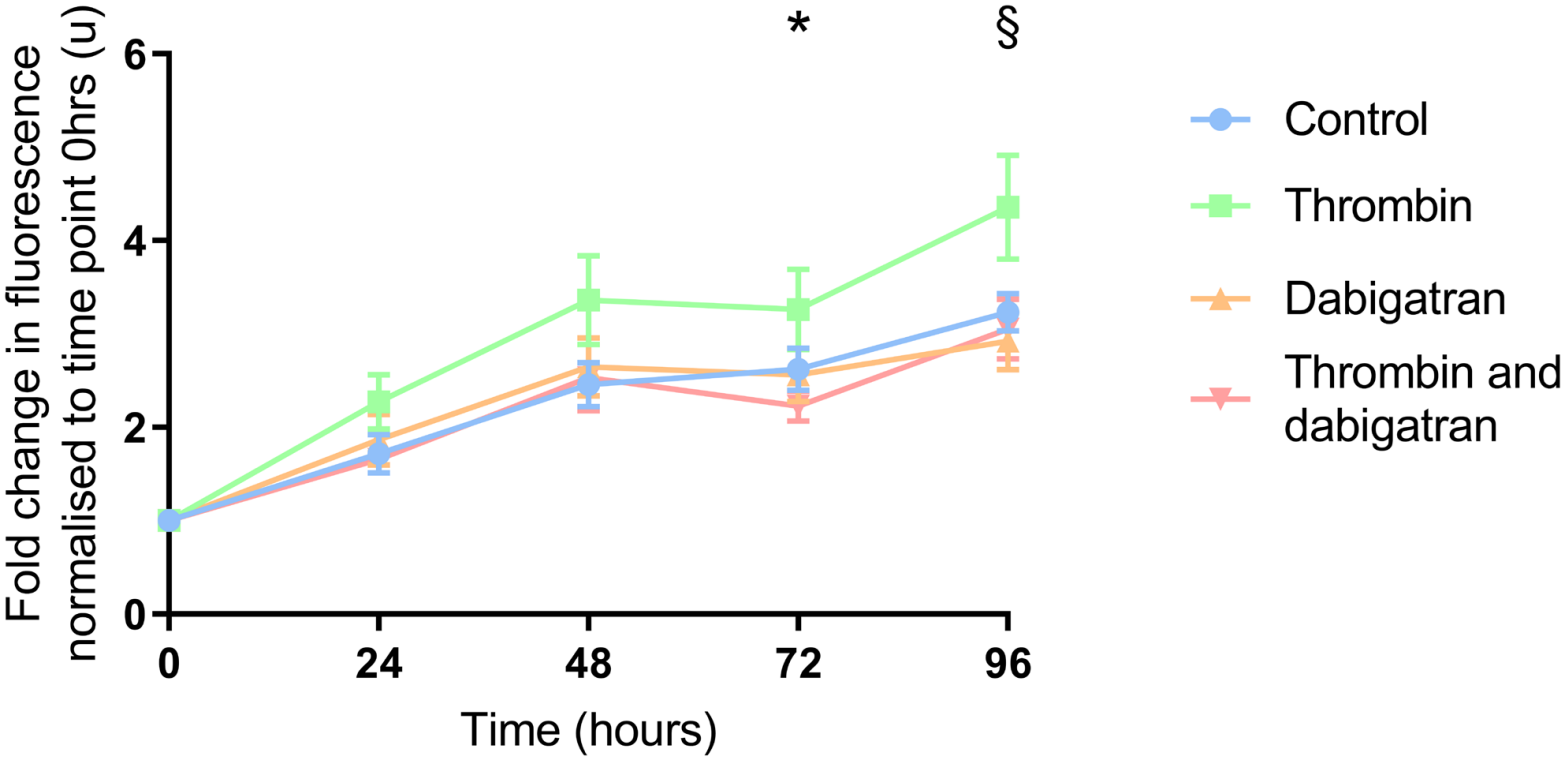
The effect of dabigatran on the pro‐proliferative effects of thrombin. SW620 cells were seeded (2.5 × 10^3^ per well). After 24 h treatment thrombin (10 U/mL) ± dabigatran (0.5 μM) was added. PrestoBlue® reagent was added at intervals and fluorescence was measured. The addition of dabigatran in combination with thrombin resulted in decreased proliferation compared to thrombin alone at 72 h. This continued at 96 h although did not reach statistical significance. Data presented as mean fold change in fluorescence normalised to time point 0 h ±SEM. Data from three independent experiments. Statistical differences were determined using unpaired *t* tests. *P* values quoted correspond to comparison between thrombin alone and thrombin in combination with dabigatran. *p < 0.05, ^§^ *p* = 0.06.

The increase in proliferation induced by 10 U/mL thrombin was attenuated by dabigatran at 72 h treatment in the SW620 cell line (*p* < 0.05, Figure 2). This trend appeared to extend to 96 h but did not quite reach statistical significance.

The addition of rivaroxaban did not reduce the pro‐proliferative effects of 1.0 U/mL FXa in the DLD‐1 cell line.

### 3.2 Migration

**FIGURE 3:**
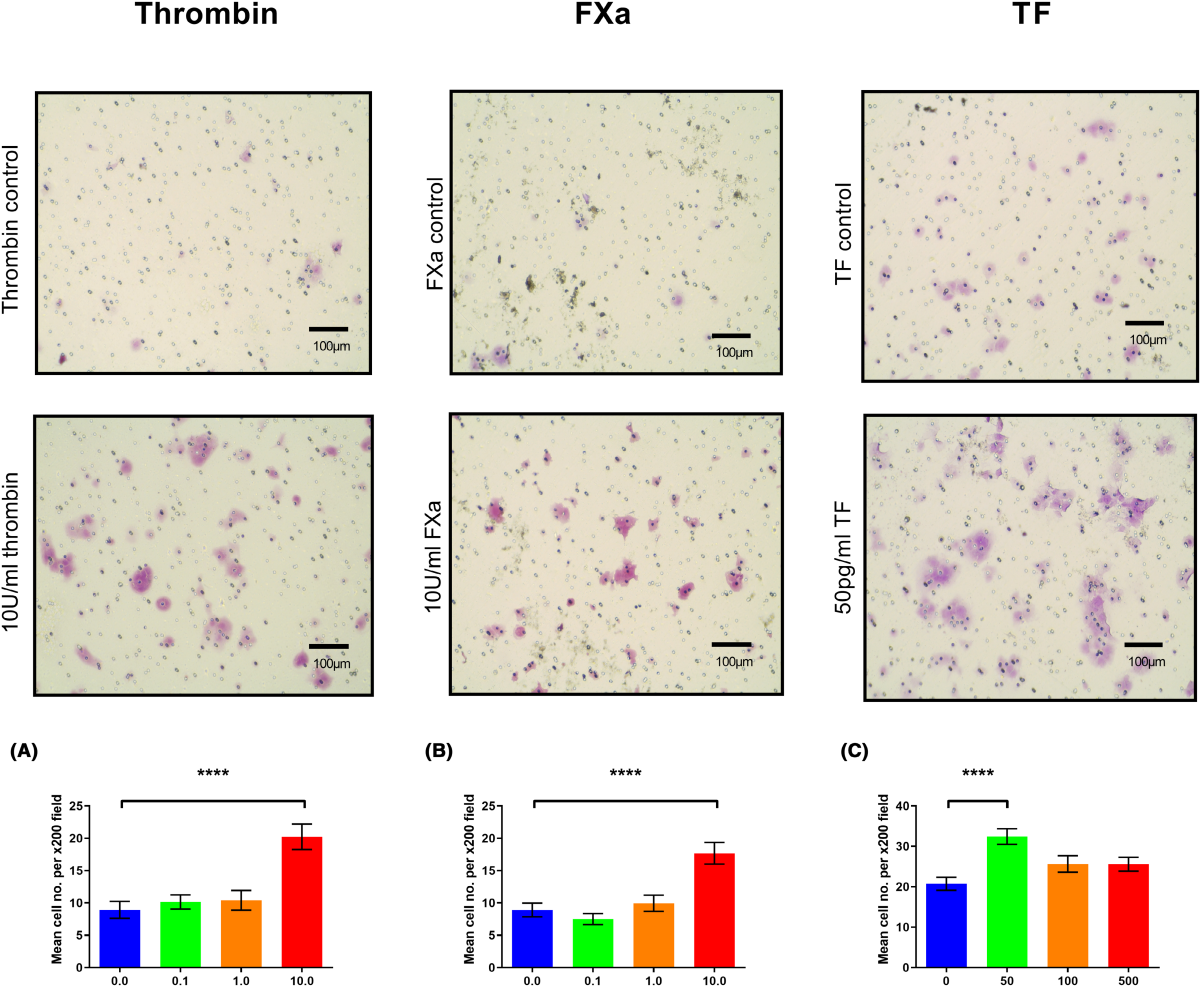
The effect of exogenous coagulation factors on migration in the DLD‐1 cell line. 5.0 × 10^4^ DLD‐1 cells were placed in the upper chamber of cell culture inserts in serum‐free conditions ±exogenous clotting factors. Media supplemented with 10% FBS was added to the lower chamber. Following incubation and staining with crystal violet the number of migrating cells were quantified with light microscopy. (A–C) DLD‐1 cells treated with 10 U/mL of thrombin, 10 U/mL of FXa and 50 pg/mL of TF, respectively, demonstrated increased migration compared to their vehicle controls. Data presented as mean number of migrated cells per ×200 field on light microscopy ±SEM. Data from three independent experiments. Statistical differences were determined using analysis of variance (ANOVA). ****p < 0.0001. Pictures demonstrate representative examples of ×200 views seen on light microscopy for vehicle controls and relevant concentrations of exogenous clotting factors. TF, tissue factor.

**FIGURE 4:**
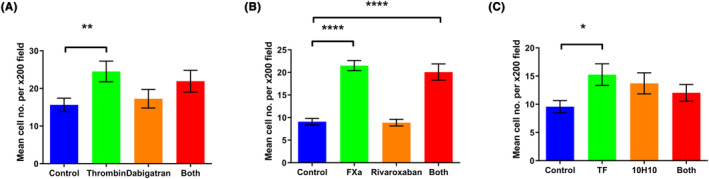
The effect of direct inhibitors on the pro‐migratory effects of exogenous clotting factors in DLD‐1 cells. 5.0 × 10^4^ DLD‐1 cells were placed in the upper chamber of cell culture inserts in serum‐free conditions ± exogenous clotting factors ± direct inhibitors. Media supplemented with 10% FBS was added to the lower chamber. Following incubation and staining with crystal violet the number of migrating cells were quantified with light microscopy. (A–C) The pro‐migratory effects of thrombin, FXa and TF were not attenuated by their respective inhibitors. Data presented as mean number of migrated cells per ×200 field on light microscopy ±SEM. Data from three independent experiments. Statistical differences were determined using independent Student's *t*‐tests. *p < 0.05, **p < 0.01, ****p < 0.0001. TF, tissue factor.

Thrombin (10.0 U/mL, Figure 3A), FXa (10.0 U/mL, Figure 3B) and TF (50 pg/mL, Figure 3C) all increased migration in the DLD‐1 cell line. However, the addition of dabigatran, rivaroxaban and 10H10 did not abrogate the pro‐migratory effects of their respective coagulation factors (Figure 4).

## 4 DISCUSSION

Evaluation of cancer cell lines by deep learning of multiomics datasets has predicted SW620 to be a particularly good model for colorectal cancer.^28^ The ability of 10 U/mL thrombin to increase proliferation among SW620 colorectal cancer cells is supported by the findings of Darmoul et al. who reported increased in vitro proliferation in colon cancer cell lines not including SW620 after 96 h of ~4 U/mL thrombin treatment.^29^ Interestingly, SW620 has a relatively low level of PAR‐1 (*F2R*) mRNA expression compared to DLD‐1 but much higher levels of endogenous thrombin (*F2*) mRNA expression.^30^ It is therefore possible that the combined endogenous thrombin generation of SW620 cells plus exogenous thrombin are together sufficient to produce the increased proliferation observed.

Similarly, the increase in cell proliferation observed in DLD‐1 colorectal cancer cells treated with 1.0 U/mL FXa may in part be explained by the coagulation factor expression profile of these cells. DLD‐1 has higher levels of tissue factor expression than SW620^14^ that with the addition of its FXa co‐factor may have produced a pro‐proliferative response via PAR‐2 (*F2RL1*), which is also expressed in higher levels in DLD‐1 versus SW620.^30^ The lack of increased proliferation at the higher FXa concentration of 10 U/mL may reflect previous observations that stimulation of cancer cell proliferation by coagulation factors forms a bell‐shaped curve, with higher concentrations less effective at increasing growth.^31^

The absence of a pro‐proliferative response in both SW620 and DLD‐1 cells treated with tissue factor could indicate that the experiment concentrations based on colorectal cancer patient plasma levels (50–500 pg/mL^27^) were too low. We based the tissue factor concentrations on plasma levels as the marked increase in tissue factor expression in colorectal tumour epithelium and stroma compared to surrounding normal cells makes it very difficult to accurately quantify concentrations in tissue.^32^ Clinically, a speculated pro‐proliferative response at a higher tissue factor level could indicate a change from a migrating phenotype to a proliferating one at a metastatic site where tissue factor levels are higher. However, the in vitro work of Zhou et al. using SW620 and our own unpublished work using breast cancer cells strongly suggests that simultaneous treatment of Factor VIIa alongside tissue factor is required to increase proliferation via PAR‐2.^33^ The higher gene expression of *F2RL1* (PAR‐2) in colorectal carcinoma chemotherapy non‐responders compared to responders in the publically available ROC Plotter resource may be an indication of pro‐proliferative signalling via TF/FXa/FVIIa.^34^, ^35^

The abrogation of a thrombin‐induced increase in proliferation in SW620 cells by a clinically relevant dabigatran concentration is to our knowledge the first report of this direct oral anticoagulant (DOAC) having an anti‐cancer effect in colorectal cancer. Graf et al. have reported a reduction in the in vivo tumour growth of MC38 mouse colon adenocarcinoma cell tumours using rivaroxaban. However, they ascribed this result to rivaroxaban blocking a reduction in anti‐tumour immunity caused by FXa‐producing myeloid cells, as evidenced by further reduction in tumour growth observed in checkpoint inhibitor plus rivaroxaban combination.^19^ Our in vitro experiments do not recapitulate the colorectal cancer immune microenvironment, but the anti‐proliferative effect we report here may support the case for further examination of dabigatran as an anti‐cancer agent in colorectal cancer. A recent retrospective study observed colorectal cancer patients on dabigatran anticoagulation having lower risk of cancer‐related death compared to CRC patients on rivaroxaban.^36^ The authors suggested this may be due to strong expression of thrombin in colorectal cancer, as reported in our previous work.^32^ The high endogenous expression of *F2* (thrombin) by SW620 supports this mechanism.

As far as we are aware, the increase in migration in DLD‐1 cells treated with 10 U/mL thrombin is the first report of this functional effect in a mismatch repair deficient (MMRd) colorectal cancer model. MMRd is present in ~15% of colorectal cancers and is treated with immune checkpoint inhibitors in the metastatic setting.^37^ This finding strengthens the case to combine direct oral anticoagulants with immune checkpoint inhibitors as proposed by Graf et al.^19^ Previously, increased migration in MMR proficient HT29‐D4 and SW480 human colon adenocarcinoma cells treated with ~2 and 0.5 U/mL thrombin, respectively, has been reported.^29^, ^38^ SW480 cells have a higher migratory potential than SW620 cells,^39^ which may explain the lower pro‐migratory thrombin concentrations previously observed.^38^ Adams et al. have demonstrated using an in vivo MC38 murine colon adenocarcinoma cell model that prothrombin promoted local invasion and was a major determinant of metastatic potential.^24^ Our previous finding that thrombin and tissue factor are expressed in the stroma of colorectal cancer tumours suggests a possible source of a pro‐migratory thrombin stimuli.^32^

We found that low concentration 0.1 U/mL FXa treatment did not affect DLD‐1 migration within our results, (8.9 vs. 7.5 cells per ×200 field, n.s.) aligning with a possible inhibitory effect of FXa on colorectal cancer cell line migration at low concentrations previously reported.^40^ However, high concentration 10 U/mL FXa treatment resulted in an almost twofold increase in DLD‐1 migration compared to control, and although clinical colorectal cancer FXa levels remain undetermined, this high concentration may better model the CRC hypercoagulable state. Given FXa is directly involved in thrombin generation and thrombin is pro‐migratory,^29^, ^38^ clinically we would hypothesise that higher FXa levels lead directly/indirectly to increased colorectal cancer cell migration.

Our finding that 50 pg/mL tissue factor increases DLD‐1 cell migration may be the first report of a stimulatory effect of exogenous TF in in vitro colorectal cancer models. Interestingly, we have found ~50 pg/mL tissue factor to be the mean plasma concentration in colorectal cancer patients awaiting curative surgery (unpublished data from our group). This may indicate colorectal cancer cells such as DLD‐1 receive a pro‐migratory tissue factor stimuli when metastasising through the circulation. Furthermore, tissue factor‐positive microparticles are present in higher levels in advanced colorectal cancer patient plasma than in age‐ and sex‐matched controls.^41^ Tian et al. have reported that tissue factor expression in colon cancer cell lines including DLD‐1 is correlated with their invasive ability and TF knockdown in LoVo colon cancer cells reduces invasion/migration in vitro and hepatic metastasis in vivo through downregulation of matrix metalloproteinases MMP2 and MMP9.^42^ As tissue factor positive microparticles and their inherent procoagulant activity may be transferred to cancer cells,^43^, ^44^ this presents another mechanism by which colorectal cells could obtain increased migratory/invasive properties in the tumour microenvironment/circulation, although we have not explored this here.

Surprisingly, none of the investigated direct inhibitors were able to abrogate the pro‐migratory effects of the exogenous clotting factors during these experiments or reduce endogenous DLD‐1 cell migration levels. We used concentrations of dabigatran and rivaroxaban based on standard clinical post‐dose plasma levels (0.5 μM and 100 ng/mL, respectively), but it may be that higher concentrations would have reduced cell motility in our model. Increased doses could be a clinically effective anti‐migration treatment, but this would have to have to be investigated in clinical trials with clinical endpoints, balancing the potential anti‐migration effect with the known side effects, the foremost being increased bleeding risk. Given the high interest of tissue factor‐targeting antibody drug conjugates in colorectal cancer, interrogation of a potential anti‐metastatic effect remains of clinical interest.

## 5 CONCLUSIONS

In conclusion, the addition of exogenous coagulation factors promote the key cellular processes of migration and proliferation in vitro in CRC cell lines. Of particular note, dabigatran appears to abrogate the pro‐proliferative effects of thrombin at a clinically relevant dose. This raises the exciting prospect of this commonly used anticoagulant as a potential anti‐cancer therapy.

## AUTHOR CONTRIBUTIONS

**Peter Adam Rees:** Data curation (lead); formal analysis (lead); investigation (lead); methodology (lead); project administration (lead); visualization (lead); writing – original draft (lead). **John Castle:** Data curation (supporting); project administration (supporting); supervision (supporting); visualization (supporting); writing – review and editing (lead). **Hamish William Clouston:** Investigation (supporting); methodology (supporting); project administration (supporting); supervision (supporting). **Rebecca Lamb:** Formal analysis (supporting); investigation (supporting); methodology (supporting); project administration (supporting); resources (supporting); supervision (equal). **Urvashi Singh:** Writing – review and editing (supporting). **Sarah Elizabeth Duff:** Conceptualization (equal); funding acquisition (equal); resources (equal); supervision (equal). **Cliona Clare Kirwan:** Conceptualization (equal); funding acquisition (equal); resources (equal); supervision (equal).

## Data Availability

The datasets generated and analysed in this research article are available in the Figshare repository [DOI 10.48420/22787444].
